# Ultrasound in Rheumatologic Interstitial Lung Disease: A Case Report of Nonspecific Interstitial Pneumonia in Rheumatoid Arthritis

**DOI:** 10.1155/2015/107275

**Published:** 2015-07-09

**Authors:** A. Laria, A. Lurati, M. Scarpellini

**Affiliations:** Rheumatology Unit, Fornaroli Hospital Magenta, 20013 Magenta, Italy

## Abstract

According to the American Thoracic Society (ATS)/European Respiratory Society consensus classification, idiopathic interstitial pneumonias (IIPs) include several clinic-radiologic-pathologic entities: idiopathic pulmonary fibrosis (IPF), usual interstitial pneumonia (UIP), nonspecific interstitial pneumonia (NSIP), cryptogenic organizing pneumonia, acute interstitial pneumonia, respiratory bronchiolitis-associated ILD, desquamative interstitial pneumonia, and lymphoid interstitial pneumonia. Ultrasound Lung Comets (ULCs) are an echographic chest-sonography hallmark of pulmonary interstitial fibrosis. We describe the ultrasound (US) findings in the follow-up of a NSIP's case in rheumatoid arthritis (RA).

## 1. Introduction

Rheumatoid arthritis is characterized by extra-articular manifestations such as lung involvement that include interstitial pneumonia and fibrosis, rheumatoid (necrobiotic) nodules, bronchiectasis, obliterative bronchiolitis (BOOP), follicular bronchiolitis, and pleural effusion or thickening [1–4]. Interstitial fibrosis has been reported in about 40% of patients who have rheumatoid arthritis; however the majority of these patients have a chest radiography normal, while evidence of interstitial fibrosis is seen at chest radiography and at high-resolution CT in only 5% and 30%–40% of patients with RA, respectively [3–6]. Rheumatoid lung disease is more frequent in men between 50 and 60 years of age [7]. NSIP can be distinguished from UIP by temporal uniformity in fibrotic processes; it seems that NSIP pattern has a better prognosis than for the UIP pattern [8, 9]. Bouros et al. reported that NSIP was the predominant pathologic pattern in scleroderma, while it is known that UIP is predominant in RA [10, 11]. However NSIP could be present also in RA and sometimes it can anticipate appearance of joint symptoms [11]. In the early stage, the radiographic appearance consists of irregular linear hyperattenuating areas in a fine reticular pattern. The abnormality usually involves mainly the lower lung zones. With the progression of disease, the reticular pattern becomes more coarse and diffuse, and honeycombing may be seen [12]. The predominant abnormality at high-resolution CT (HR-CT) consists of irregular linear hyperattenuating areas caused by a combination of intralobular lines and irregular thickening of interlobular septa. Honeycombing is seen. Interstitial lung changes are frequent and independent of disease duration. Interstitial changes are more frequent and severe in rheumatoid factor-positive patients and in patients with more severe joint involvement [13]. Chest HR-CT is actually considered as the diagnostic gold standard to assess pulmonary fibrosis. Ultrasound Lung Comets (ULCs, a recently described echographic sign of interstitial lung fibrosis) are images of multiple comet tails fanning out from the lung surface. They originate from water-thickened or fibrosis-thickened interlobular septa. They have a significant positive linear correlation with Warrick scores of pulmonary fibrosis to high-resolution CT (HR-CT) [2]. They represent a simple, bedside, radiation-free hallmark of pulmonary fibrosis with a potential diagnostic and prognostic value. We want to describe an ultrasound (US) echographic follow-up of a clinical case of NSIP in RA.

## 2. Case Report

**Figure 1:**
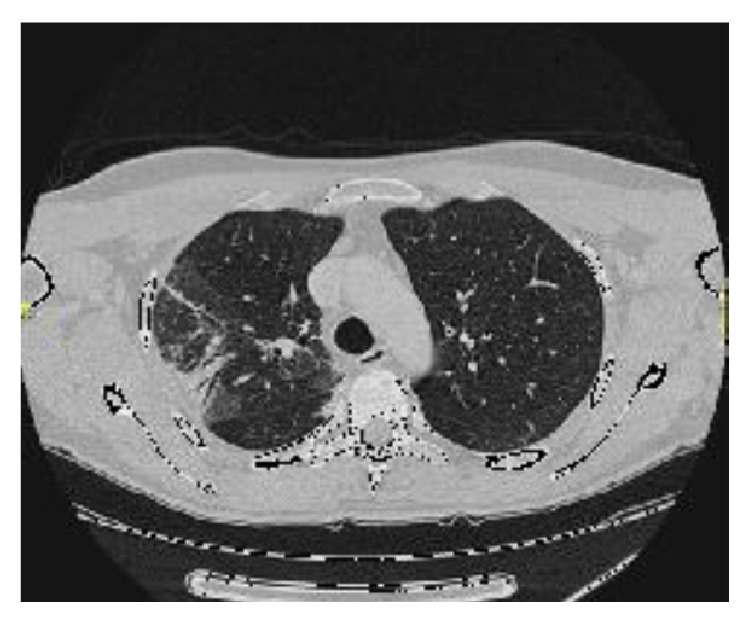
HR-CT lung pretreatment: interstitial consolidation with centrilobular branching linear structures with bronchial dilatation and multifocal areas of ground-glass attenuation at right superior lobe of lung in the posterior segment.

**Figure 2:**
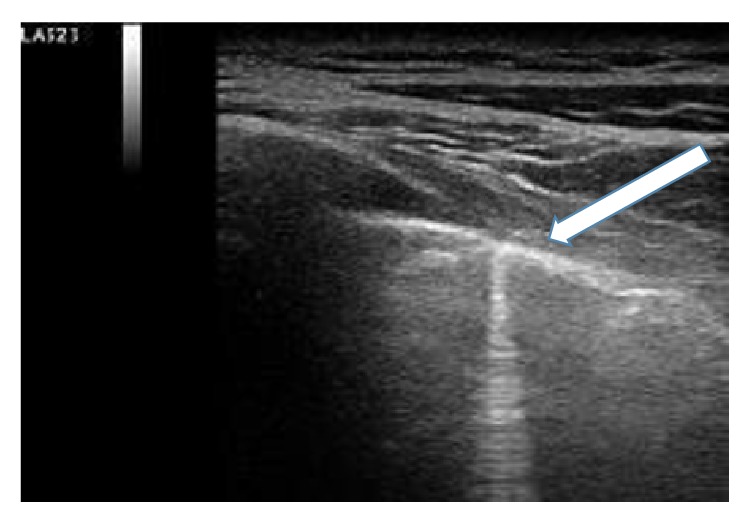
Ultrasound (US) echographic of right lung made in April 2014 before treatment. Presence of Ultrasound Lung Comets (ULCs) at the fifth right posterior intercostal space (arrow).

**Figure 3:**
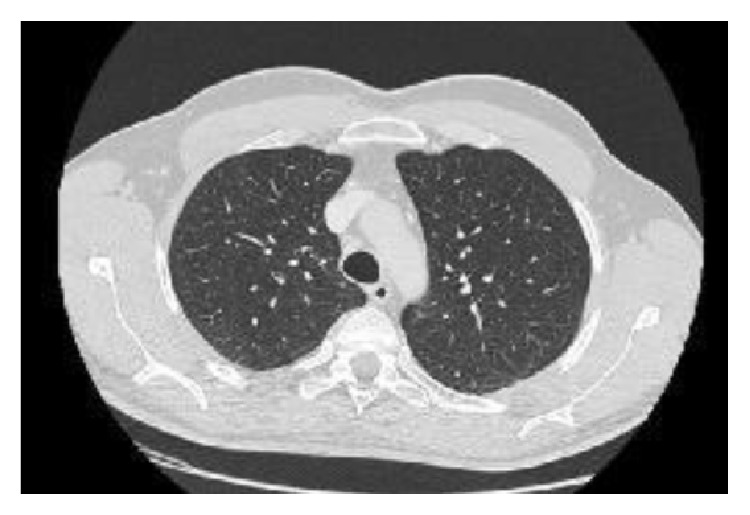
HR-CT lung pretreatment: resolution of interstitial flogistic involvement.

**Figure 4:**
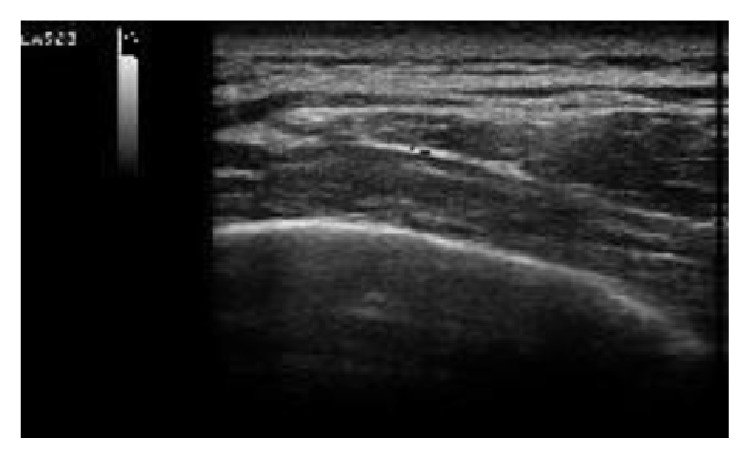
Ultrasound (US) echographic of right lung made in September 2014 after treatment. Disappearance of Ultrasound Lung Comets (ULCs) at the fifth right posterior intercostal space.

A 54-year-old male was referred to the Rheumatology Division of Fornaroli Hospital from the Infective Department. In February 2014, he was admitted to hospital because of interstitial lung disease of uncertain origin accompanied by fever with shiver, dry cough, and chest pain. Chest X-ray showed a right subclavicle interstitial consolidation. A lung/abdominal CT (Computerized Tomography) with contrast enhancement showed an interstitial consolidation with centrilobular branching linear structures with bronchial dilatation and multifocal areas of ground-glass attenuation at the right superior and medium lobe of lung in the posterior segment. There were pleura's thickening at the lingula and fibrotic lines with consolidation at the left superior lobe of lung in the anterior segment, also. Moreover, there were some mediastinic bilateral lymphadenopathies (Figure 1). Laboratory tests showed ESR 28 mm/h (cut-off value ≤10 mm/h) and C-reactive protein 6.66 mg/dL (range of normality: 0–5). Antinuclear antibodies (ANA), cryoglobulin, extractable nuclear antibodies (ENAs), anti-DNA native antibodies, antiphospholipid antibodies, and antineutrophil cytoplasmic antibody (ANCA) were negative. Complementemia (C3, C4) was at range of normality. Rheumatoid factor (RF) was 24 UI/mL (range of normality <12) and anti-cyclic citrullinated peptide (anti-CCP) antibodies were 17 UA/mL (range of normality <5). Serum angiotensin-converting enzyme (ACE) levels were normal. Quantiferon-TB Gold was negative. Microbial agents were excluded (serology for* Legionella*,* Streptococcus pneumoniae*,* Mycoplasma pneumoniae*, and* Chlamydia* was negative). A restrictive defect on functional lung exams was excluded and diffusion capacity of the lung for carbon monoxide (DLCO) was normal. Smoking history was absent. A lung bronchoscopy with a bronchoalveolar lavage (BAL) was made. Bacterial BAL fluid cultures were negative. Culture and genome for Koch's bacillus were negative. BAL cytology excluded monoclonality disorders and showed increased numbers of neutrophils and eosinophils. Lung CT showed multifocal areas of ground-glass and we hypothesized a rheumatologic versus infective interstitial lung disease. So, lung biopsy was not performed in this patient because we think that it was excessive forasmuch as BAL was performed (including cultures and cytology) which supported inflammatory diagnosis. A positron emission tomography-computed tomography (^18^F-FDG PET/CT) showed an elevated glucometabolic activity at bilaterally axillary lymphadenopathies and at level of pulmonary consolidation seen at HR-CT; these abnormalities were hypothesized to inflammatory nature and possible rheumatic disease (such as vasculitis). Heart ultrasound, nailfold videocapillaroscopy, and superior abdomen ultrasound did not show abnormalities. After some weeks, painful swelling of right knee appeared. Doppler ultrasound (US) of low limbs excluded deep venous thrombosis. He received a large spectrum antibiotic therapy (before, at home he had assumed oral levofloxacin therapy and later oral amoxicillin clavulanate; during the recovery he received oral doxicilin and azithromycin at standard dosage) with reduction of fever but persistence of lung abnormities to radiologic exams. So, It was started a steroid therapy ex adiuvantibus with a clinical subjective improvement. In the suspect of rheumatologic disease patient was assigned to our division. During rheumatologic recovery painful swelling of right wrist, knee, and ankle appeared. We made an ultrasound (US) echographic of the lung (Figure 2). Commercially available echographic equipment with a 7.5 MHz linear probe was used (Mylab25, Esaote, Genoa, Italy). US of right lung, at the fifth posterior intercostal space, showed the presence of Ultrasound Lung Comets (ULCs) (as echographic sign of interstitial lung involvement). On the basis of clinical features and laboratory/functional/radiologic tests we made a diagnosis of NSIP in RA seropositive for RF. We started immunosuppressive therapy with intravenous cyclophosphamide 1 gr every 28 days for a cumulative dose of 6 gr (from 3 April to 10 September 2014) associated with steroid tapering therapy. We observed any adverse events during treatment with cyclophosphamide. At the end of intravenous infusion of cyclophosphamide, a lung high-resolution CT was performed. It showed a significant improvement of interstitial lung disease with disappearance of multifocal areas of ground-glass attenuation and consolidations (Figure 3). Also ultrasound evaluation at the end of therapy showed complete resolution of ULCs (Figure 4). So we decided to stop other infusions of cyclophosphamide and to start treatment with methotrexate 10 mg/weekly for arthralgia of right wrist as well as maintenance therapy associated with low dose of steroids (prednisone 5 mg/day tapering).

## 3. Discussion

Rheumatoid arthritis is a chronic inflammatory autoimmune disease that targets joints but can involve other organs such as the lung. Positivity for RF and/or anti-CCP increases risk to develop interstitial lung disease that in majority of cases appears later to joint involvement. ULCs are images of multiple comet tails fanning out from the lung surface. They originate from water-thickened or fibrosis-thickened interlobular septa. In the presence of extravascular lung water, such as in the presence of fibrosis in the parietal pleura or interlobular septa, the ultrasound beam finds subpleural interlobular septa thickened by oedema. The reflection of the beam creates a phenomenon of reverberation [14]. Differential diagnosis of ULCs originating from the pleural line has been made. Cardiogenic ULCs occur in greater numbers than pneumogenic ones. Cardiogenic ULCs are more prevalently localized in the right lung than in the left one, with a “hot zone” of higher density in the right anterior-axillary third intercostal space. Cardiogenic ULCs disappeared after diuretic therapy. Instead, pneumogenic ULCs are present equally in the lungs with a distribution more patchy without response to diuretic therapy [14, 15]. Our clinical case report supports data literature that in few cases interstitial pulmonary precedes appearance of arthritis and that in few cases rheumatoid lung is characterized by NSIP pattern (not UIP one). Our patient did not have a lung biopsy performed. So we cannot assert if ULCs were related to lung fibrosis or subpleural interlobular septa thickened by oedema. However, good and fast response to medical therapy indirectly related the ULCs with an acute lung injury rather than lung fibrosis. Ultrasound lung echographic (US) follow-up is more reproducible than HR-CT because of bedside and radiation-free hallmarks, with a good significant correlation between ULCs and radiologic score to HR-CT. So the potential diagnostic and prognostic value of ultrasound (US) lung echographic appear to be evident.
